# Use of Contact Networks to Estimate Potential Pathogen Risk Exposure in Hospitals

**DOI:** 10.1001/jamanetworkopen.2022.25508

**Published:** 2022-08-05

**Authors:** Kaniz Fatema Madhobi, Ananth Kalyanaraman, Deverick J. Anderson, Elizabeth Dodds Ashley, Rebekah W. Moehring, Eric T. Lofgren

**Affiliations:** 1 School of Electrical Engineering and Computer Science, Washington State University, Pullman; 2 Duke Center for Antimicrobial Stewardship and Infection Prevention, Duke University School of Medicine, Durham, North Carolina; 3 Paul G. Allen School for Global Health, Washington State University, Pullman

## Abstract

**Importance:**

Person-to-person contact is important for the transmission of health care–associated pathogens. Quantifying these contact patterns is crucial for modeling disease transmission and understanding routes of potential transmission.

**Objective:**

To generate and analyze the mixing matrices of hospital patients based on their contacts within hospital units.

**Design, Setting, and Participants:**

In this quality improvement study, mixing matrices were created using a weighted contact network of connected hospital patients, in which *contact* was defined as occupying the same hospital unit for 1 day. Participants included hospitalized patients at 299 hospital units in 24 hospitals in the Southeastern United States that were part of the Duke Antimicrobial Stewardship Outreach Network between January 2015 and December 2017. Analysis was conducted between October 2021 and February 2022.

**Main Outcomes and Measures:**

The mixing matrices of patients for each hospital unit were assessed using age, Elixhauser Score, and a measure of antibiotic exposure.

**Results:**

Among 1 549 413 hospitalized patients (median [IQR] age, 44 [26-63] years; 883 580 [56.3%] women) in 299 hospital units, some units had highly similar patterns across multiple hospitals, although the number of patients varied to a great extent. For most of the adult inpatient units, frequent mixing was observed for older adult groups, while outpatient units (eg, emergency departments and behavioral health units) showed mixing between different age groups. Most units mixing patterns followed the marginal distribution of age; however, patients aged 90 years or older with longer lengths of stay created a secondary peak in some medical wards. From the mixing matrices by Elixhauser Score, mixing between patients with relatively higher comorbidity index was observed in intensive care units. Mixing matrices by antibiotic spectrum, a 4-point scale based on priority for antibiotic stewardship programs, resulted in 6 major distinct patterns owing to the variation of the type of antibiotics used in different units, namely those dominated by a single antibiotic spectrum (narrow, broad, or extended), 1 pattern spanning all antibiotic spectrum types and 2 forms of narrow- and extended-spectrum dominant exposure patterns (an emergency room where patients were exposed to one type of antibiotic or the other and a pediatric ward where patients were exposed to both types).

**Conclusions and Relevance:**

This quality improvement study found that the mixing patterns of patients both within and between hospitals followed broadly expected patterns, although with a considerable amount of heterogeneity. These patterns could be used to inform mathematical models of health care–associated infections, assess the appropriateness of both models and policies for smaller community hospitals, and provide baseline information for the design of interventions that rely on altering patient contact patterns, such as practices for transferring patients within hospitals.

## Key Points

**Question:**

What are the mixing patterns among hospitalized patients who could be susceptible to infection?

**Findings:**

This quality improvement study of 1 549 413 hospitalized patients in 299 hospital units from 24 hospitals analyzed the mixing patterns between patients based on age, antibiotic use by spectrum, and Elixhauser Comorbidity Score. While some units showed highly similar patterns across hospitals, there was considerable variation and evidence that patients frequently came into contact with other patients with markedly different age- and antibiotic exposure–based risk factors.

**Meaning:**

The findings of this study regarding how patients mix and the variability of that mixing among hospitals could improve models of hospital-associated infections as well as guidelines in community hospital settings.

## Teaser

This quality improvement study uses mixing matrices to describe the contact networks of hospitalized patients in a large, multihospital sample to assess contact patterns of variables associated with susceptibility to hospital-acquired infections.

## Introduction

An individual’s risk of acquiring an infectious disease is inherently a function of whom they contact, with infected individuals representing the exposure source for those infected in the future, a phenomenon known as *dependent happenings*.^1^ Understanding whom an individual contacts becomes critical for understanding their risk. One way of studying this information is as a contact network, represented by a population of individuals (nodes) and the contacts between them (edges), and studying the properties of this network.

Contact patterns in infectious diseases have been extensively studied in HIV and other sexually transmitted diseases^2,3,4,5^ and are being increasingly studied in infectious diseases more broadly.^6,7,8,9^ Hospitalized patients represent a particularly challenging population for contact network analyses owing to the complexity of the hospital environment. Patients may contact each other directly (depending on whether they are mobile), and they may be exposed to pathogens through contamination on the hands and clothes of health care workers, on shared instruments, or the hospital environment as fomites. Several studies have collected hospital contact networks using a variety of methods.^10,11,12,13^ However, many of these studies were limited to a single hospital or study site. Long-term and multisite studies of these networks may be important for understanding how hospitals adjust to shifting demands for patient care (eg, during a pandemic), the evolution of antibiotic stewardship programs, or other shifts to the flow of hospitalized patients, and how this in turn impacts infection control.

The focus of this study was to better describe the contact networks of hospitalized patients using a large, multihospital sample. By forming these contact networks, we aimed to visualize contact patterns of variables associated with susceptibility to hospital-acquired infections and multidrug resistant organisms (MDROs): age, comorbidity, hospital unit type, and antibiotic exposure. We examine age because it is a known risk factor for infectious diseases, such as COVID-19,^14^ as well as a number of health care–associated infections.^15,16,17^ We also examine Elixhauser Score^18^ for comorbidity as a proxy for overall vulnerability to infection, and antibiotic use as a measure for potential MDRO colonization pressure from other patients within the unit.^19^ We hypothesize that the mixing matrices for hospitals will have considerable between-unit and between-hospital variability.

## Methods

This quality improvement study was deemed exempt from approval and informed consent by the Duke University Health System institutional review board because it was not human participants research. Analyses were conducted between October 2021 and February 2022.

### Patient Data

To estimate the patient contact networks, we used data from the Duke Antimicrobial Stewardship Outreach Network^20^ and Duke Health System, which contain curated hospital encounter records for 24 community hospitals and 1 academic medical center in the Southeastern United States between October 2015 and December 2017.

### Network Estimation

Within the data, there are records for a patient’s movement between units, as well as arrival and discharge. We estimated a colocation contact network, ie, if 2 patients were recorded as being in the same hospital unit during a period of 1 day, they were counted as being in contact for that day. If there are *k* patients in a unit on a specific day, the number of contacts would be *k choose 2 (kC2)*. For example, 4 patients (A, B, C, and D) located in the same unit on a specific day will imply 6 (ie, *4C2*) pairwise contacts: A and B, A and C, A and D, B and C, B and D, and C and D. As a patient may be admitted to 1 or more hospitals, each patient admission was considered to be a unique node in the assembled network.

It is important to note that the type of unit is based on its National Healthcare Safety Network classification. This classification provides a useful, but imperfect, approximation of patient mix, as patients may be placed in a unit for many reasons, such as bed availability and hospital volume, or a unit’s definition may shift over time. To quantify these networks, network-level measures were collected, including diameter, mean degree, mean betweenness, mean closeness, modularity, and density. The association between these measurements and hospital size was assessed using generalized linear models. The numbers of edges emerging from patients who had single-ward stays vs multiward stays were calculated.

### Computation of Mixing Matrices

Using the pairwise contact information, we computed 3 types of mixing matrices, based on patient age, Elixhauser Score, and antibiotic agent exposure. These mixing matrices record the frequency of contacts between patients belonging to different classes of that category.

For analysis of mixing by age, we constructed a 2-dimensional table in which the patient’s age (range, 0-90 years) was represented by the rows and columns. Each cell (eg, i, j) corresponds to the number of patient contacts between a patient of age *i* with a patient of age *j*.

Elixhauser Score or Elixhauser Comorbidity Index is a measure of patient comorbidity, developed in 1998.^18^ In Duke Antimicrobial Stewardship Outreach Network data, the Elixhauser Score ranged from 0 to 16, with a higher score indicating a greater degree of comorbid conditions in a patient. The mixing matrices in this case are 16 × 16 tables in which the value in a cell (eg, i, j) corresponds to the frequency of contact between a patient having comorbidity score *i* with a patient having comorbidity score *j*.

For mixing matrices by antibiotic agents, we followed a ranking scheme proposed by Moehring et al.^21^ Antibiotic agents are categorized on a 4-point scale based on their spectrum of activity and priority for antibiotic stewardship programs as follows: narrow spectrum, broad spectrum, extended spectrum, and protected. The resulting mixing matrices become 4 × 4 tables in which the value in a cell (eg, i, j) represents the number of contacts occurring between patient pairs exposed to agent ranks *i* and *j* respectively.

This method accounts for both the distribution of the relevant variable of interest (ie, age, Elixhauser Score, or antibiotic use) as well as the role of time. For example, a small group of individuals with long lengths of stay (LOS) would create a hotspot when the mixing matrix is visualized disproportionate to methods that simply considered the distribution of the variables without weighting by LOS.

To help with comparisons between different hospital sizes, we also computed a normalized representation for the mixing matrices by scaling each cell as follows:

*Normalized Contacts* = (*Unnormalized Contacts* − *Minimum Contacts*) / (*Maximum Contacts* − *Minimum Contacts*)

### Statistical Analysis

Differences in distributions were tested for statistical significance using χ^2^ tests, or Fisher exact test in the presence of small sample sizes. Linear regression models were used to explore the association between. All significance tests were 2-sided, with a significance threshold of α = .05. Statistical analysis was performed using the R statistical language version 4.2.0 (R Project for Statistical Computing).

Data preparation and network extraction were performed using Python version 3.6.9 (Python Software Foundation), including the Bokeh^22^ library for visualization and the creation of interactive plots. These interactive plots allow for comparing the mixing matrices across different units, and across different hospitals and are available online.^23^ The extracted patient contact networks, as well as source code, are also available online.^24^

## Results

### Sample Characteristics

Our data included 1 549 413 hospitalized patients (median [IQR] age, 44 [26-63] years; 883 580 [56.3%] female patients) in 299 total units across all hospitals, ranging from 4 units at the smallest hospital to 30 units at the largest hospital. There was a median (IQR) of 62 992 (36 078-86 482) patients per hospital. The Table shows the basic statistics and corresponding demographic characteristics.

**Table.** Basic Statistics of the DASON Database and Corresponding Demographic Information

Attribute	Value
**Wards**	
Total across 24 hospitals, No.	299
Per hospital, median (IQR)	11 (9-15)
**Admissions**	
Total across 24 hospitals, No.	2 903 357
Per hospital, median (IQR)	128 291 (70 633-162 441)
**Patients**	
Total across 24 hospitals, No.	1 569 413
Per hospital, median (IQR)	62 992 (36 078-86 482)
Age, median (IQR) [range], y	44 (26-63) [0-90]
Elixhauser Score, median (range)	0 (0-16)
Sex, No. (%)	
Female	883 580 (56.3)
Male	685 833 (39.8)
Missing	61 207 (3.9)

### Patient Contact Networks

In eFigure 1 in the Supplement, we added a snapshot of the patient contact networks that were generated using the records of 1 month (January 2017) for each of the hospitals included in this study. The nodes represent patients, and edges represent a pairwise contact between the corresponding 2 patients in the time interval considered. The largest network in this collection (for hospital 24) had 10 957 nodes, and the mean (SD) degree (ie, edges connecting to that node; in this case, the number of colocated patients) was 300.3 (208.7); while the smallest network (for hospital 16) had 642 nodes and a mean (SD) degree of 32.0 (21.2). Hospital size was negatively associated with density, which decreased by 0.00016 (95% CI, −0.000079 to −0.00024) per 100 patients (*P* < .001). Hospital size was positively associated with mean degree, which increased by 3.00 (95% CI, 2.64 to 3.36) per 100 patients (*P* < .001) and mean betweenness, which increased by 84.08 (95% CI, 75.0 to 93.2) per 100 patients (*P* < .001). The full network level measurements is presented in eTable 1 in the Supplement. The percentage of patients with multiple unit stays ranged from 0% (hospital 16) to 65.0% (hospital 8), and the percentage of contacts arising from multiple unit stays ranged from 0% (hospital 16) to 93.3% (hospital 8). Neither were associated with hospital size. Hospital-level details are presented in eTable 2 in the Supplement.

### Mixing Matrix by Age

There was considerable interhospital variability in the age-mixing patterns of patients, owing to myriad factors, such as the type of hospital and catchment population (eFigure 2 in the Supplement). There were primarily 3 major patterns observed: (1) hospitals showing uniform mixing across all adult ages (eg, hospital 23), (2) hospitals serving primarily younger age groups (eg, hospital 11), and (3) hospitals, especially smaller ones, dominated by mixing among elderly patients (eg, hospital 5). These mixing patterns of hospitals were consistent with their respective age distributions (eFigure 3 in the Supplement).

**Figure 1.**
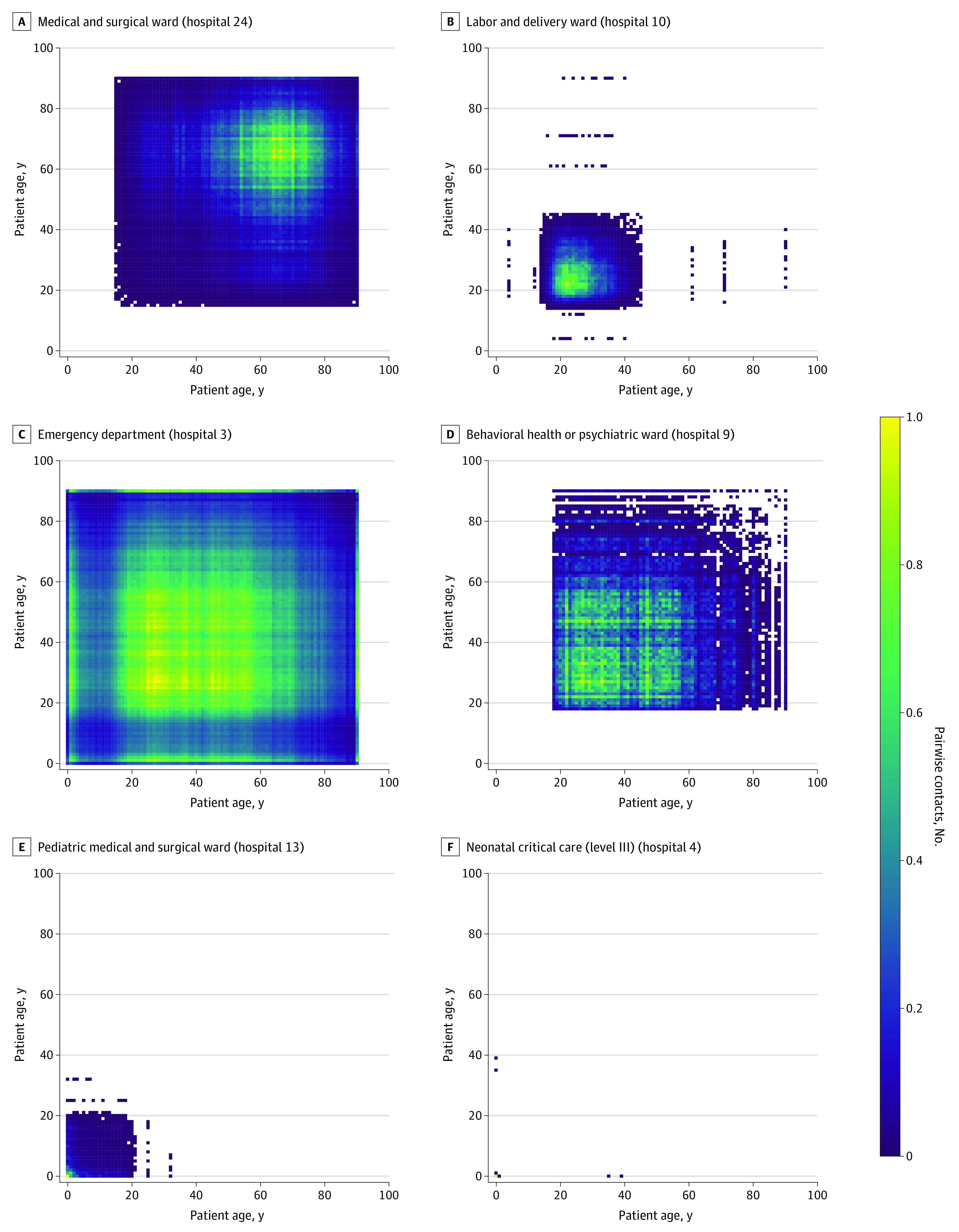
Mixing Matrices by Age Measured by the Number of Pairwise Contacts for Selected Single Hospital Units Small numbers of incongruous patients (eg, patients in labor and delivery wards aged 60 years or older) represent small amounts of misclassification, either in patient demographics or the National Healthcare Safety Network unit type as a proxy for what a ward is being used for but are retained for completeness. Darker shades indicate lower mixing; brighter shades, heavier mixing.

However, within a hospital, the patterns varied from unit to unit, as different units cater to different types of patients. Figure 1 shows a selected subset of 6 mixing matrices drawn from different hospital units. Most of the adult inpatient units (eg, medical and surgical [Figure 1A]) featured a larger number of patients aged 40 years and older, with a high density of patients approximately 65 years of age, with few young patients. However, because the mixing matrices also account for LOS, these broad peaks centered on patients aged approximately 65 years are part of a bimodial distribution, with a second, sharp peak for patients aged 90 years and older, where the peak was not driven by the total number of patients in that age group but by particularly long LOS (eFigure 4 in the Supplement).

Labor and delivery wards similarly showed robust patterns between hospitals, concentrated among patients aged 20 to 35 years, tapering off toward the limits of maternal age (Figure 1B). The mixing patterns for some other units, such as operating room suites and 24-hour observation, showed wide variation among hospitals. Outpatient units, such as emergency departments (Figure 1C) and behavioral health units (Figure 1D) had mixing among broader age ranges, with a larger proportion of the mixing among patients in the age range of 20 to 50 years. In addition, the ED also showed mixing between adult and child age groups. Pediatric units (Figure 1E) showed broad mixing of patients younger than 20 years, although concentrated in infants. Neonatal units (Figure 1F) were dominated by mixing among infants and young adults of childbearing age.

### Mixing Matrix by Elixhauser Score

**Figure 2.**
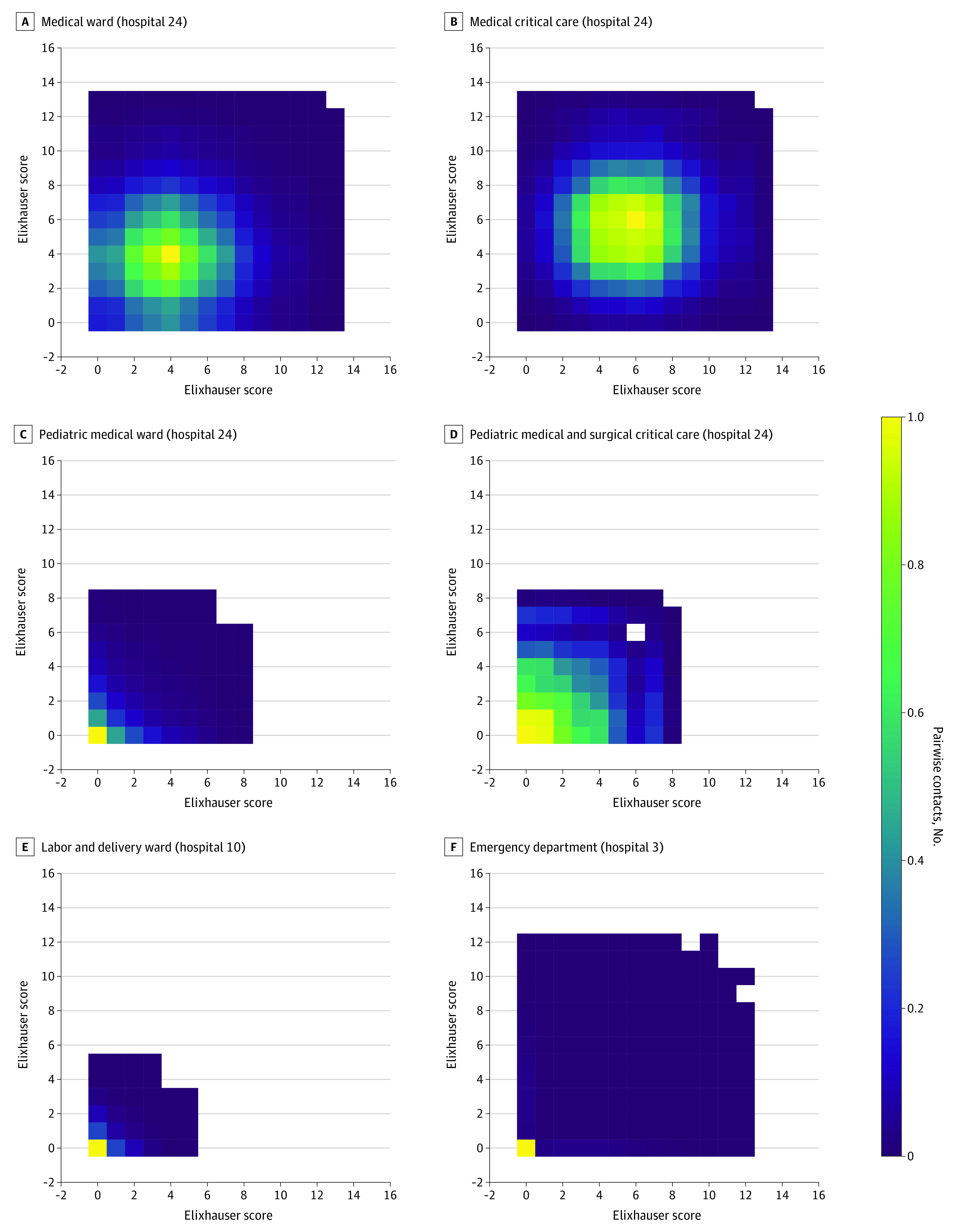
Mixing Matrices by Elixhauser Score Measured by the Number of Pairwise Contacts for Selected Single Hospital Units Darker shades indicate lower mixing; brighter shades, heavier mixing.

Elixhauser Scores in the data ranged from 0 to 16, with a median (IQR) 0 (0) owing to most patients having no known comorbidities. Figure 2 shows the mixing matrices by Elixhauser Score for a selected subset of 6 units taken from different hospitals. Patients in adult medical wards generally had mixing among patients with low to moderate Elixhauser Scores, with the highest density being patients with a score of 4 mixing with other patients with a score of 4; Elixhauser Scores greater 10 were exceedingly rare (Figure 2A). Adult critical care units (Figure 2B) shifted this distribution up and to the right. Noncritical (Figure 2C) and critical (Figure 2D) pediatric wards had distributions much more skewed toward patients with no comorbid conditions, albeit with a broader range of possible values in critical care units. Neonatal units were primarily concentrated at 0. Labor and delivery wards (Figure 2E) and emergency departments (Figure 2F) had characteristically low rates of comorbid conditions, but emergency departments demonstrated a wider potential range.

### Mixing Matrix by Antibiotic Category

From medication data, we found that there were many patients who were not receiving any form of antibiotic, including some wards in which most patients were not. The ratio of contacts in which neither patient in a connected pair were receiving antibiotics compared with pairs in which 1 patient or both patients were receiving antibiotics varied widely by unit (eg, 4 units presented in eFigure 5 in the Supplement). A visualization of these data for all units is available online.^23^

**Figure 3.**
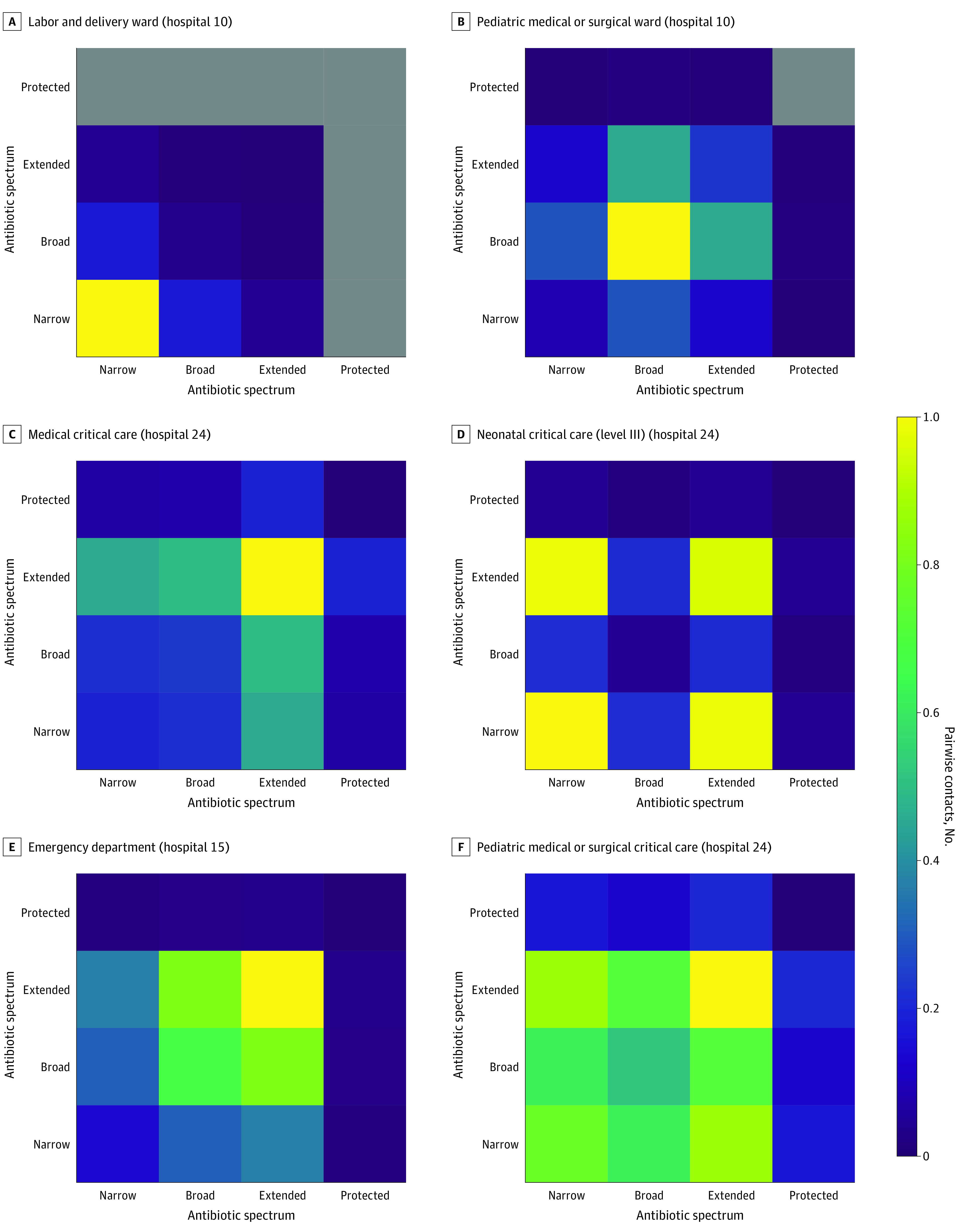
Mixing Matrices by Antibiotic Spectrum Measured by the Number of Pairwise Contacts for Selected Single Hospital Units Darker shades indicate lower mixing; brighter shades, heavier mixing.

We observed 6 distinct patterns on the antibiotic mixing matrices based on the 4-point ranking scheme (Figure 3). Across all hospitals, gynecology, labor and delivery, and postpartum units predominantly involved patients receiving narrow-spectrum agents (Figure 3A). This pattern also occurred in some, but not all, operating room suites and orthopedic wards, driven by prophylactic and postoperative cefazolin, respectively. Broad-spectrum heavy antibiotic use patterns appeared primarily in pediatric medical surgical wards (Figure 3B). Wards heavily using extended-spectrum agents (Figure 3C) were most often adult critical care units of all types. This pattern was also observed in some hospitals in postcritical care units, endoscopy suites, and 24-hour observation areas. Figure 3D shows a distinctive heavy mixing pattern for narrow- and extended-spectrum antibiotics that appeared predominantly in pediatrics-focused units, including well baby nurseries, step-down neonatal nurseries, and neonatal critical care units and also in a subset of cardiac catheterization units, surgical cardiothoracic critical care units, and neurological critical care units. A pattern dominated by broad- and extended-spectrum antibiotics (Figure 3E) was most often seen in emergency departments, as well as occasionally in some telemetry wards and 24-hour observation areas. A final pattern, involving the relatively frequent use of narrow-, broad-, and extended-spectrum antibiotics (Figure 3F) was only observed in the pediatric units of the large academic medical center, discordant with the pediatric units of the community hospitals. A visualization of these unit-specific mixing patterns for all 299 units in the data set is available elsewhere.^23^

Matrices that showed dense mixing on 1 spectrum were an indicator of substantial use of a specific kind of antibiotic in a unit. On the other hand, the bimodal interspectrum mixing could have arisen from 1 of 2 possible mechanisms: 2 distinct groups of patients, one receiving one type of antibiotic, the other receiving another type of antibiotic, who happened to be colocated in the same unit. The second is that the same patients are prescribed drugs of 2 different spectrums. To examine these 2 possibilities, we considered 1 ward with both many patients as well as the distinctive narrow- and extended-spectrum antibiotics pattern, a neonatal intensive care unit in a large academic medical center. The distribution of antibiotic exposures is shown in eTable 3 in the Supplement. It is apparent that most patients were exposed to both classes of antibiotics during their hospitalization, although the result was not statistically significant (eTable 3 and eTable 4 in the Supplement). In contrast, the analysis of a unit (emergency department in hospital 15) with a pattern of broad- and extended-spectrum antibiotics found a different result (eTable 4 in the Supplement). This unit served a large elderly population, including several skilled nursing facilities, and among them, 77.6% of patients were prescribed either broad-spectrum (33.2%) or extended-spectrum (44.4%) agents (*P* < .001).

## Discussion

In this quality improvement study, the number of contact patterns that were either more diffuse (in the case of age and Elixhauser Score) or unique (in the case of some antibiotic prescribing patterns in pediatric units) to the academic medical center highlights the level of heterogeneity among hospitals. This heterogeneity is also apparent in the properties of the hospital-level contact networks themselves, which were often, but not always, independent of the overall size of the hospital.

### Mixing Matrix by Age

Mixing matrices by age mostly conformed to expected patterns in that they reflect the marginal age distribution for the unit (with some notable exceptions due to age-associated LOS). Emergency departments and behavioral units in particular showed areas with broader age-related mixing patterns. These units require special consideration when considering pathogens with markedly different age-related risks, transmission potentials, or vaccination status.

### Mixing Matrix by Elixhauser Score

Compared with general units, the dense area in critical care units shifted up and to the right, indicating an increase in patients with prevalent comorbid conditions, although the peak for this distribution was more diffuse. Patients in the pediatric and neonatal units are not likely to have developed comorbid conditions, which explains the dense region at 0, although pediatric critical care has a broader range of possible values, reflecting their more complex patient mixture. However, given that there are several problems with the use of the Elixhauser Score in pediatric care (children having less time to acquire the diagnosis of a comorbid condition), we caution against the interpretation of these results beyond the overall notion of the broader spread of values in pediatric critical care. The emergency department, although concentrated around zero comorbidities, demonstrated mixing among a wide potential range, reflecting its central role as a possible place for patients with vastly different underlying characteristics to encounter one another.

### Mixing Matrix by Antibiotic Category

The differing patterns for mixing by antibiotic category between the neonatal intensive care unit and the emergency department is indicative of how 2 very different clinical settings can create ostensibly similar mixing patterns at the ward level, but that differ on the individual level. The neonatal intensive care unit pattern seems likely to arise from a commonly used combination of ampicillin and gentamicin for empirical coverage of neonatal sepsis.^25^ This, in turn, suggests that a patient’s individual antibiotic exposure profile likely represented their primary exposure at any given time.

In contrast, the results from the emergency department are due to a large array of antimicrobial agents being administered to different patients, and once again highlight the importance of emergency departments as areas with far broader mixing patterns than the rest of the hospital environment, as well as the likelihood of the aforementioned units being central points of empirical therapy within many hospitals.

Mixing of patients receiving narrow-, broad-, and extended-spectrum antibiotics was only observed in the pediatric units of the large academic medical center. The academic medical center is notable for having patients with cystic fibrosis, as well as a pediatric transplant program, which resulted in a markedly different patient profile compared with community hospital pediatric units and likely drove these different patterns.

### Limitations

There are limitations to this study, in part arising from the use of an existing data source to reach multiple hospitals and many patients. This study implicitly assumes that patients visiting a unit the same day had contact—primarily via indirect contact mediated by health care workers or the environment. While age, underlying comorbidities, and antibiotic exposure are important risk factors for health care–associated infections, they are not an exhaustive list, and there are a number of risk factors that are beyond the reach of a single study of this type, or impractical to collect on an ongoing basis in a broad network of hospitals of varying resource levels. Patients who occupy the same unit for multiple days have repeated contacts, which we assume linearly add to the amount of mixing. Additionally, we assume that an National Healthcare Safety Network unit designation is an adequate proxy for the type of procedures and patients present in a given unit, which may result in a degree of misclassification

## Conclusions

This quality improvement study presents several aspects of how hospitalized patients could come into contact with each other. Understanding these contact patterns can provide vital information on infection transmission risk, such as where patients with high potential susceptibility to acquiring MDROs might be in contact, directly or indirectly, with patients at serious risk for adverse outcomes from infection. While some of these patterns may be inferred heuristically, it is nevertheless beneficial to quantify these patterns. This enables their use in modeling studies of hospital-acquired pathogens. Furthermore, it could provide a means to quantitatively track shifts in antibiotic use or patient case mix patterns. Quantifying the variability between hospitals can help assess the intervention and policy recommendations in the academic medical centers where these estimates are often obtained as well as in the rural and community hospitals, for any situation where patient-to-patient interaction is potentially at play. This study highlights the need for data and parameter estimates from both academic medical centers and community and provides a broad range of estimates from hospitals of diverse sizes and catchment areas.

## Notes

Supplement. **eFigure 1.** A Snapshot of Patient Contact Networks Constructed for the DASON Data for January 2017 **eFigure 2.** Mixing Matrices by Age Measured by the Number of Pairwise Contacts for Each of 24 Community Hospitals in the DASON Network **eFigure 3.** Histograms Depicting the Distributions of the Patient Ages Included in the DASON Data Set For October 2015 to November 2017 **eFigure 4.** Patient Age Distributions Compared With Patient Length of Stay Distributions **eFigure 5.** Ratio of Contacts in Which the Neither, 1, or Both Contacts Were Receiving Antibiotics **eTable 1.** Hospital-Wide Network Statistics From a Snapshot of Patient Contact Networks Constructed From the DASON Data for January 2017 **eTable 2.** Hospitalwide Distribution of Single vs Multiple Unit Nodes and Edges From a Snapshot of Patient Contact Networks Constructed From the DASON Data for January 2017 **eTable 3.** Distribution of Patients in a Neonatal Critical Care Unit (Hospital 24) Based on Their Exposure to Narrow- and/or Extended-Spectrum Antibiotics **eTable 4.** Distribution of Patients in an Emergency Department (Hospital 15) Based on Their Exposure to Broad- and/or Extended-Spectrum Antibiotics
