# Supplementary material for: Use of Contact Networks to Estimate Potential Pathogen Risk Exposure in Hospitals
Source: JAMA Netw Open. 2022 Aug 5;5(8):e2225508. doi: 10.1001/jamanetworkopen.2022.25508 (PMC9356318; doi:10.1001/jamanetworkopen.2022.25508)
Supplement: Supplement. — eFigure 1. A Snapshot of Patient Contact Networks Constructed for the DASON Data for January 2017 eFigure 2. Mixing Matrices by Age Measured by the Number of Pairwise Contacts for Each of 24 Community Hospitals in the DASON Network eFigure 3. Histograms Depicting the Distributions of the Patient Ages Included in the DASON Data Set For October 2015 to November 2017 eFigure 4. Patient Age Distributions Compared With Patient Length of Stay Distributions eFigure 5. Ratio of Contacts in Which the Neither, 1, or Both Contacts Were Receiving Antibiotics eTable 1. Hospital-Wide Network Statistics From a Snapshot of Patient Contact Networks Constructed From the DASON Data for January 2017 eTable 2. Hospitalwide Distribution of Single vs Multiple Unit Nodes and Edges From a Snapshot of Patient Contact Networks Constructed From the DASON Data for January 2017 eTable 3. Distribution of Patients in a Neonatal Critical Care Unit (Hospital 24) Based on Their Exposure to Narrow- and/or Extended-Spectrum Antibiotics eTable 4. Distribution of Patients in an Emergency Department (Hospital 15) Based on Their Exposure to Broad- and/or Extended-Spectrum Antibiotics [file jamanetwopen-e2225508-s001.pdf]

Supplemental Online Content

Madhobi KF, Kalyanaraman A, Anderson DJ, Dodds Ashley E, Moehring RW, Lofgren ET. Use of contact networks to estimate potential pathogen risk exposure in hospitals. *JAMA Netw Open*. 2022;5(8):e2225508. doi:10.1001/jamanetworkopen.2022.25508

eFigure 1. A Snapshot of Patient Contact Networks Constructed for the DASON Data for January 2017
eFigure 2. Mixing Matrices by Age Measured by the Number of Pairwise Contacts for Each of 24 Community Hospitals in the DASON Network
eFigure 3. Histograms Depicting the Distributions of the Patient Ages Included in the DASON Data Set For October 2015 to November 2017
eFigure 4. Patient Age Distributions Compared With Patient Length of Stay Distributions
eFigure 5. Ratio of Contacts in Which the Neither, 1, or Both Contacts Were Receiving Antibiotics
eTable 1. Hospital-Wide Network Statistics From a Snapshot of Patient Contact Networks Constructed From the DASON Data for January 2017
eTable 2. Hospitalwide Distribution of Single vs Multiple Unit Nodes and Edges From a Snapshot of Patient Contact Networks Constructed From the DASON Data for January 2017
eTable 3. Distribution of Patients in a Neonatal Critical Care Unit (Hospital 24) Based on Their Exposure to Narrow- and/or Extended-Spectrum Antibiotics
eTable 4. Distribution of Patients in an Emergency Department (Hospital 15) Based on Their Exposure to Broad- and/or Extended-Spectrum Antibiotics

This supplemental material has been provided by the authors to give readers additional information about their work.

© 2022 Madhobi KF et al. *JAMA Network Open.*

eFigure 1. A Snapshot of Patient Contact Networks Constructed for the DASON Data for January 2017

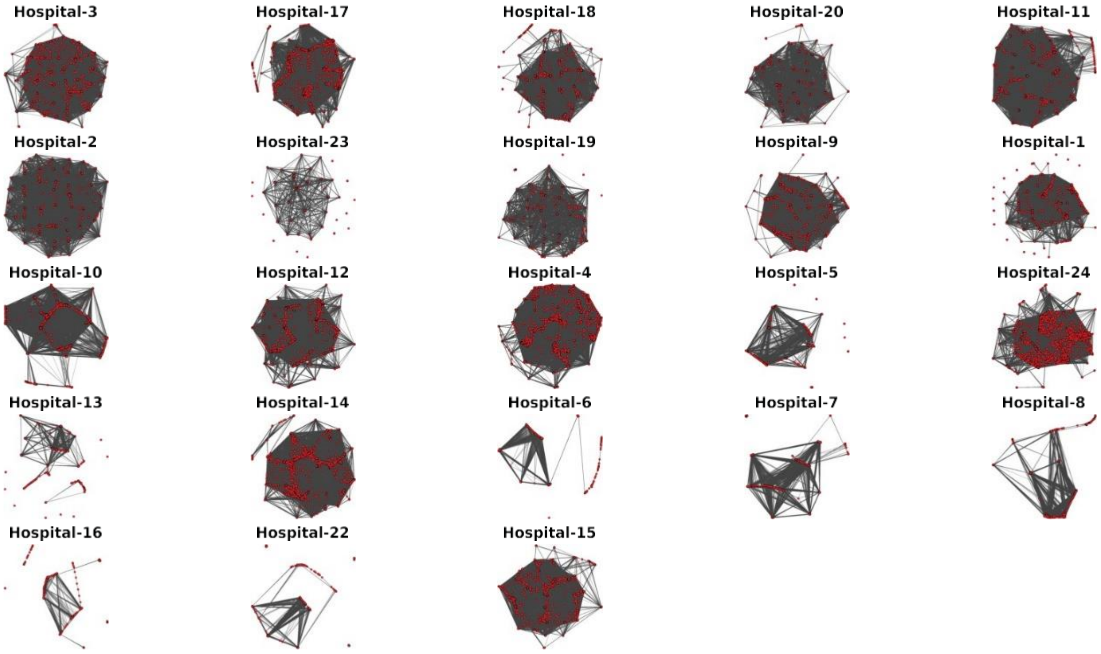

Each node is a patient, and each edge represents a contact between the two corresponding patients in that hospital during that month. One hospital is omitted due to a very sparse connectivity over the chosen month.

© 2022 Madhobi KF et al. *JAMA Network Open.*

eFigure 2. Mixing Matrices by Age Measured by the Number of Pairwise Contacts for Each of 24 Community Hospitals in the DASON Network

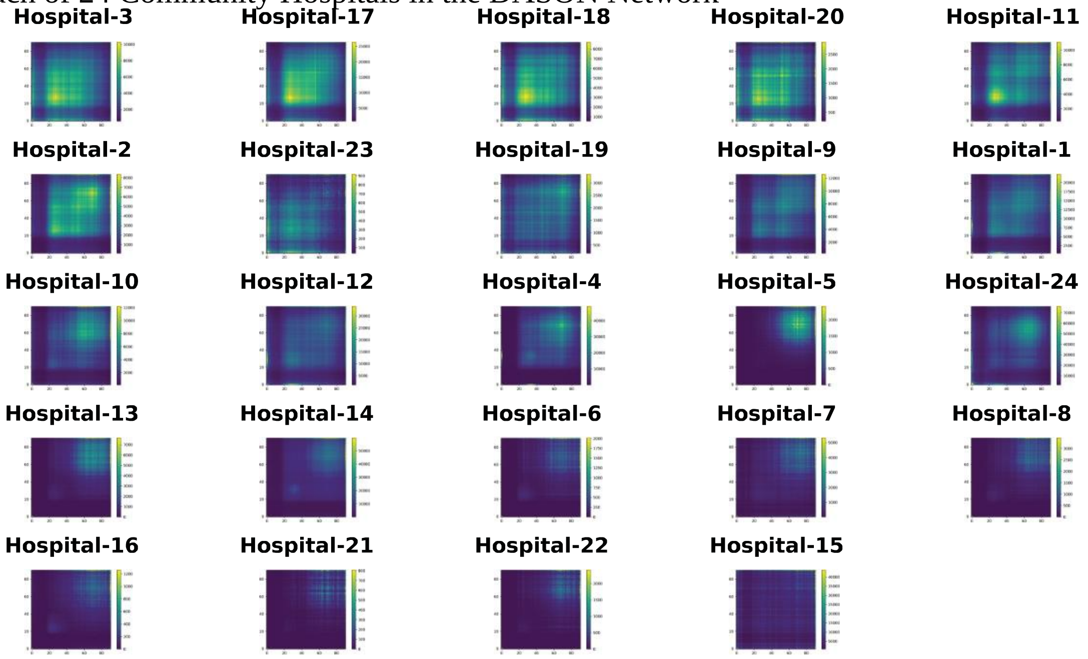

Brighter areas of color represent denser numbers of patient-to-patient connections based on occupation in the same unit. Each hospital is represented on its own scale.

© 2022 Madhobi KF et al. *JAMA Network Open.*

eFigure 3. Histograms Depicting the Distributions of the Patient Ages Included in the DASON Data Set For October 2015 to November 2017

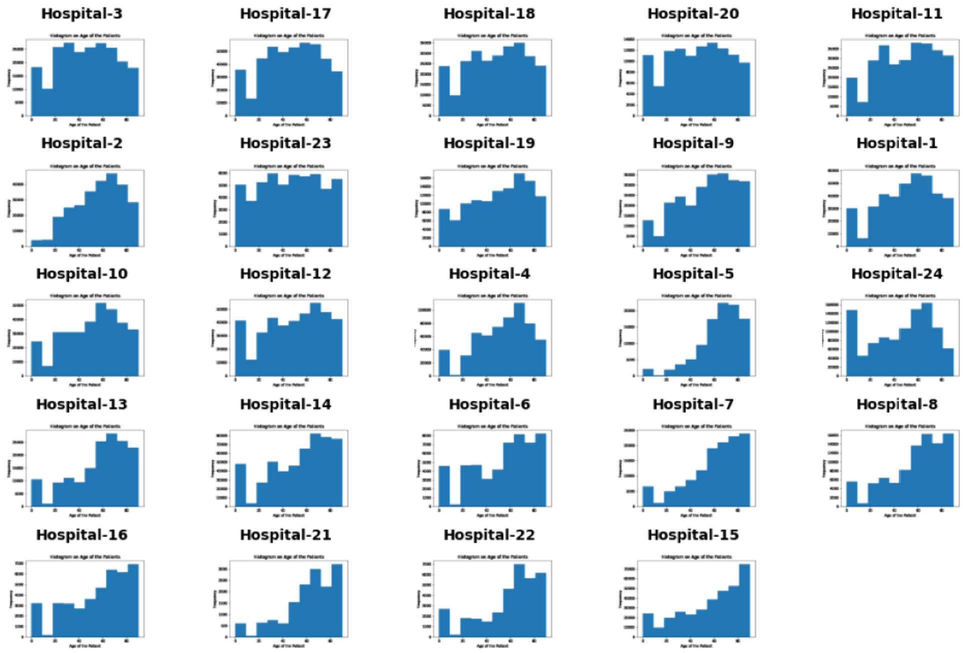

eFigure 4. Patient Age Distributions Compared With Patient Length of Stay Distributions

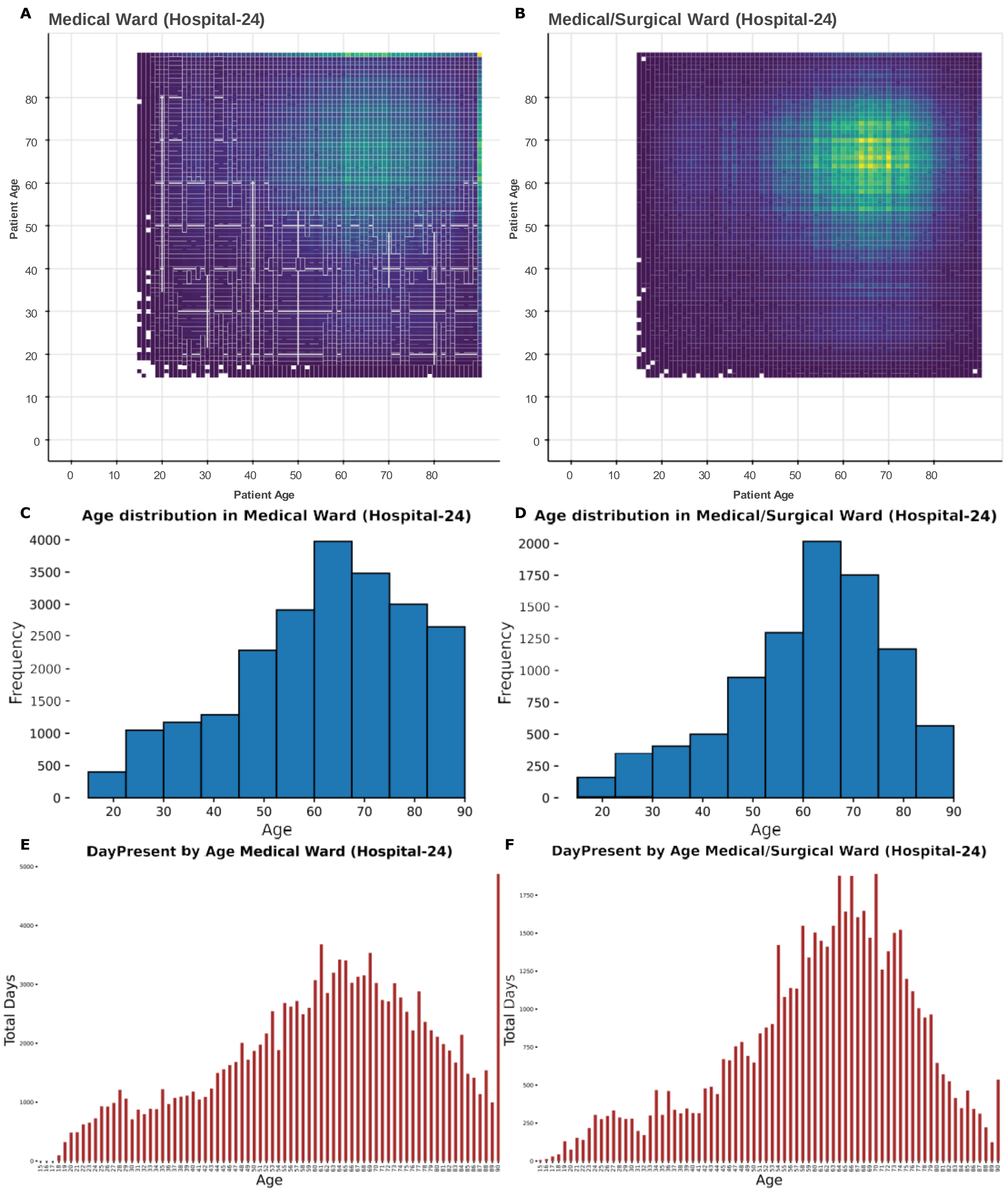

Patient groups with particularly long lengths of stay contribute disproportionately to the mixing matrix as compared to looking at the age distributions alone.

© 2022 Madhobi KF et al. *JAMA Network Open.*

eFigure 5. Ratio of Contacts in Which the Neither, 1, or Both Contacts Were Receiving Antibiotics

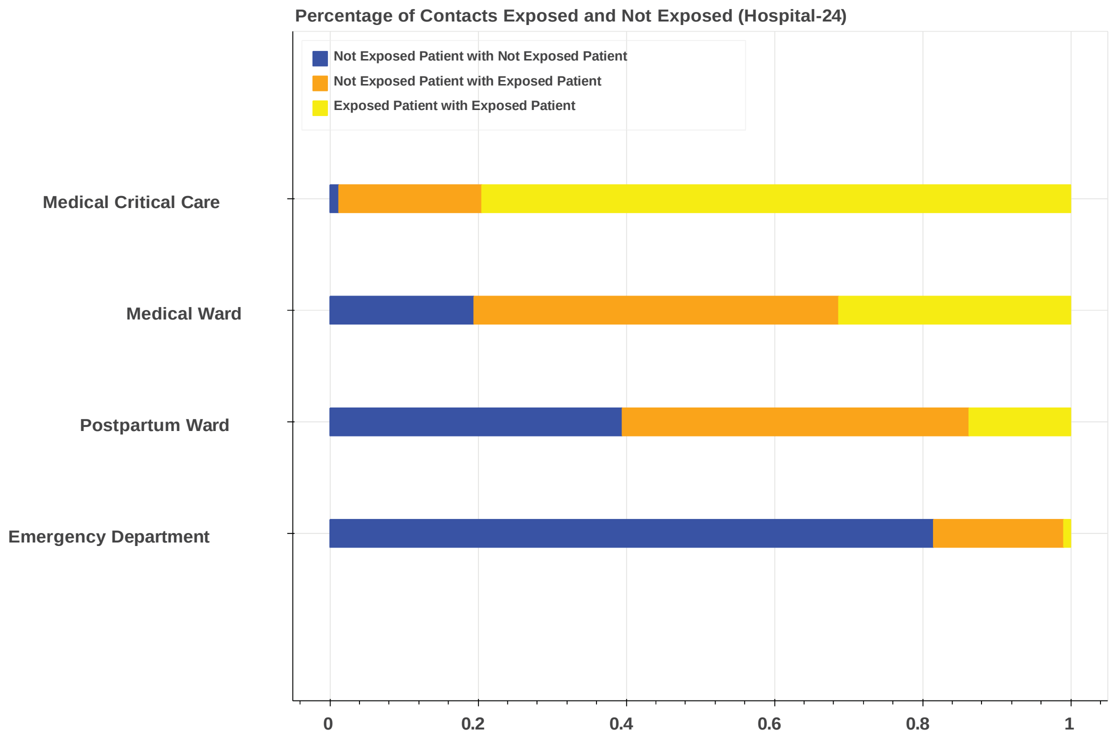

Here, the top two barcharts show a comparative scenario in a Medical Ward and a Medical Critical Care from the same hospital. While medical care has a higher ratio on one of the patients on antibiotics, the critical care is apparently higher in the ratio for both of the patients on antibiotics. The barchart from Postpartum Ward shows the ratio where almost half of the contacts were between patients who were not on any antibiotic and for the remaining half is distributed amongst the other two categories. And the last is drawn from the Emergency Department which shows most of the contacts occurred in this unit were between patients who were not exposed to any antibiotic until that point.

eTable 1. Hospital-Wide Network Statistics From a Snapshot of Patient Contact Networks Constructed From the DASON Data for January 2017

Hospital ID	Nodes	Edges	Diameter	Mean Degree	Mean Betweenness	Mean Closeness	Modularity	Density
1	6576	670913	6	204.049	5356.133	0.385	0.764	0.016
2	5389	401977	5	149.184	4982.137	0.354	0.801	0.014
3	7269	894488	13	246.110	7631.447	0.320	0.821	0.017
4	7657	874364	7	228.383	6689.637	0.370	0.668	0.015
5	1122	26168	5	46.645	760.520	0.408	0.666	0.021
6	729	17407	15	47.887	800.048	0.302	0.631	0.033
7	1177	31604	10	53.748	935.178	0.380	0.698	0.023
8	1074	34933	17	65.113	1379.834	0.312	0.559	0.030
9	5882	602574	8	204.887	5191.893	0.355	0.762	0.017
10	3539	246644	7	139.386	2902.438	0.369	0.641	0.020
11	6255	620917	8	198.535	6415.586	0.334	0.781	0.016
12	7598	1094890	6	288.205	6895.834	0.359	0.761	0.019
13	1573	62816	12	79.969	1007.687	0.375	0.595	0.025
14	7309	919447	6	251.593	5072.496	0.395	0.668	0.017
15	5810	710865	16	244.704	3575.345	0.462	0.684	0.021
16	642	10271	16	32.047	160.092	0.386	0.736	0.025
17	8810	1578582	6	358.361	7630.618	0.358	0.769	0.020
18	5837	607447	8	208.137	5458.206	0.347	0.796	0.018
19	2841	145634	7	102.523	3054.066	0.319	0.802	0.018
20	3979	311686	9	156.665	4190.498	0.319	0.799	0.020
22	723	25099	9	69.430	460.033	0.424	0.519	0.048
23	1418	37640	6	53.089	1625.326	0.318	0.834	0.019
24	10957	1645446	10	300.346	8521.860	0.392	0.673	0.014

© 2022 Madhobi KF et al. *JAMA Network Open.*

eTable 2. Hospitalwide Distribution of Single vs Multiple Unit Nodes and Edges From a Snapshot of Patient Contact Networks Constructed From the DASON Data for January 2017

Hospital ID	Nodes	Edges	Single Unit Nodes (%)	Multi-Unit Nodes (%)	Single Unit Edges (%)	Multi-Unit Edges (%)
1	6576	670913	5222 (79.4)	1354 (20.6)	375799 (56.0)	295114 (44.0)
2	5389	401977	4254 (78.9)	1135 (21.1)	222959 (55.5)	179018 (44.5)
3	7269	894488	6392 (87.9)	877 (12.1)	649383 (72.6)	245105 (27.4)
4	7657	874364	4955 (64.7)	2702 (35.3)	300864 (34.4)	573500 (65.6)
5	1122	26168	901 (80.3)	221 (19.7)	11909 (45.5)	14259 (54.5)
6	729	17407	606 (83.1)	123 (16.9)	12529 (72.0)	4878 (28.0)
7	1177	31604	997 (84.7)	180 (15.3)	17658 (55.9)	13946 (44.1)
8	1074	34933	376 (35.0)	698 (65.0)	2324 (6.7)	32609 (93.3)
9	5882	602574	4393 (74.7)	1489 (25.3)	282160 (46.8)	320414 (53.2)
10	3539	246644	1434 (40.5)	2105 (59.5)	21117 (8.6)	225527 (91.4)
11	6255	620917	4808 (76.9)	1447 (23.1)	351592 (56.6)	269325 (43.4)
12	7598	1094890	6392 (84.1)	1206 (15.9)	684951 (62.6)	409939 (37.4)
13	1573	62816	1211 (77.0)	362 (23.0)	35555 (56.6)	27261 (43.4)
14	7309	919447	5486 (75.1)	1823 (24.9)	344566 (37.5)	574881 (62.5)
15	5810	710865	4396 (75.7)	1414 (24.3)	375720 (52.9)	335145 (47.1)
16	642	10271	642 (100)	0 (0)	10271 (100)	0 (0)
17	8810	1578582	7406 (84.1)	1404 (15.9)	1023232 (64.8)	555350 (35.2)
18	5837	607447	4843 (83.0)	994 (17.0)	412821 (68.0)	194626 (32.0)
19	2841	145634	2492 (87.7)	349 (12.3)	103399 (71.0)	42235 (29.0)
20	3979	311686	3360 (84.4)	619 (15.6)	206008 (66.1)	105678 (33.9)
22	723	25099	606 (83.8)	117 (16.2)	12745 (50.8)	12354 (49.2)
23	1418	37640	1333 (94.0)	85 (6.0)	31504 (83.7)	6136 (16.3)
24	10957	1645446	7403 (67.6)	3554 (32.4)	652800 (39.7)	992646 (60.3)

© 2022 Madhobi KF et al. *JAMA Network Open.*

eTable 3. Distribution of Patients in a Neonatal Critical Care Unit (Hospital 24) Based on Their Exposure to Narrow- and/or Extended-Spectrum Antibiotics

	Narrow-spectrum exposed	Narrow-spectrum unexposed
Extended-spectrum exposed	1,230	9
Extended-spectrum unexposed	44	2

eTable 4. Distribution of Patients in an Emergency Department (Hospital 15) Based on Their Exposure to Broad- and/or Extended-Spectrum Antibiotics

	Took broad-spectrum	Did not take broad-spectrum
Took extended-spectrum	2,445	6,499
Did not take extended-spectrum	4,856	833

© 2022 Madhobi KF et al. *JAMA Network Open.*
